# Antiproliferative and Antimicrobial Activities of Selected Bryophytes

**DOI:** 10.3390/molecules23071520

**Published:** 2018-06-23

**Authors:** Martin Vollár, András Gyovai, Péter Szűcs, István Zupkó, Marianna Marschall, Boglárka Csupor-Löffler, Péter Bérdi, Anikó Vecsernyés, Attila Csorba, Erika Liktor-Busa, Edit Urbán, Dezső Csupor

**Affiliations:** 1 Department of Pharmacognosy, Faculty of Pharmacy, University of Szeged, H-6720 Szeged, Hungary; vollar@pharmacognosy.hu (M.V.); csupor.boglarka@pharmacognosy.hu (B.C.-L.); veasaat.sze@gmail.com (A.V.); csorba@pharmacognosy.hu (A.C.); erikal@email.arizona.edu (E.L.-B.); 2 Interdisciplinary Centre for Natural Products, University of Szeged, H-6720 Szeged, Hungary; 3 Department of Pharmacodynamics and Biopharmacy, Faculty of Pharmacy, University of Szeged, H-6720 Szeged, Hungary; gyovaiandras@gmail.com (A.G.); zupko@pharm.u-szeged.hu (I.Z.); berdi.peter@pharm.u-szeged.hu (P.B.); 4 Department of Botany and Plant Physiology, Institute of Biology, Eszterházy Károly University, H-3300 Eger, Hungary; szucs.peter@uni-eszterhazy.hu (P.S.); marschall.marianna@uni-eszterhazy.hu (M.M.); 5 Institute of Clinical Microbiology, Faculty of Medicine, University of Szeged, H-6720 Szeged, Hungary; urban.edit@med.u-szeged.hu

**Keywords:** bryophytes, antiproliferative, antibacterial, MTT assay

## Abstract

One-hundred and sixty-eight aqueous and organic extracts of 42 selected bryophyte species were screened in vitro for antiproliferative activity on a panel of human gynecological cancer cell lines containing HeLa (cervix epithelial adenocarcinoma), A2780 (ovarian carcinoma), and T47D (invasive ductal breast carcinoma) cells using the 3-(4,5-dimethylthiazol-2-yl)-2,5-diphenyltetrazolium bromide (MTT) assay and for antibacterial activity on 11 strains using the disc-diffusion method. A total of 99 extracts derived from 41 species exerted ≥25% inhibition of proliferation of at least one of the cancer cell lines at 10 μg/mL. In the cases of *Brachythecium rutabulum*, *Encalypta streptocarpa*, *Climacium dendroides*, *Neckera besseri*, *Pleurozium schreberi*, and *Pseudoleskeella nervosa*, more than one extract was active in the antiproliferative assay, whereas the highest activity was observed in the case of *Paraleucobryum longifolium*. From the tested families, Brachytheciaceae and Amblystegiaceae provided the highest number of antiproliferative extracts. Only 19 samples of 15 taxa showed moderate antibacterial activity, including the most active *Plagiomnium cuspidatum*, being active on 8 tested strains. Methicillin-resistant *Staphylococcus aureus* (MRSA) and *Staphylococcus aureus* were the most susceptible to the assayed species. This is the first report on the bioactivities of these 14 species.

## 1. Introduction

In the era of drug development based on high-throughput pharmacological screening, there is an increasing demand for molecules to be tested. Besides large synthetic libraries, natural products are of primary importance because of their pharmacological activities and high structural diversity. In the last decades, 22% of all the newly approved drug molecules have been natural product derivatives (semisynthetic), 4% have been genuine natural products, and 13% have been made by total synthesis bearing the pharmacophore of a natural product [1]. Two of the most important research topics in drug development are anticancer agents and antibiotics. According to the World Cancer Report 2014, there were approximately 14 million new cases and 8.2 million cancer-related deaths in 2012 [2]. Although in recent years several novel strategies have been uncovered for fighting cancer, the successful treatment of several types of cancer is still a challenge, and plants continue to play a major role in drug discovery, as evidenced by the number of promising new agents in clinical development [3]. The burden of microbial infections, although most devastating in developing countries, is also increasing in Western countries as a result of spreading antibiotic resistance. Having these in mind, together with the fact that 65% of antibiotics and 35% of anticancer agents registered between 1980 and 2010 were natural products or semisynthetic derivatives thereof, the investigation of possible new sources for bioactive natural products is of primary importance. 

From a phytochemical and pharmacological point of view, the most thoroughly explored taxon of the plant kingdom is vascular plants. Bryophytes (belonging to non-vascular plants) are less well studied. Although the potential presence of bioactive secondary metabolites in these species is suggested by the fact that generally bryophytes are not damaged by microorganisms, insects, or other pests [4], the phytochemistry and pharmacological profiles of the majority of species are undisclosed. The development of microscopic or genetic identification and micropropagation techniques has triggered the research into bryophytes [5].

The bryophytes, with more than 20,000 species, comprising Marchantiophyta (liverworts, ~6000 species), Bryophyta (mosses, ~14,000 species), and Anthocerotophyta (hornworts, ~300 species), can be found everywhere in the world except in the sea. In the Hungarian flora, 659 species are present, with the predominance of mosses [6]. Although not applied in human nutrition, a number of bryophytes have been widely used as medicinal plants, particularly in China for various illnesses, including for diseases of bacterial origin [7]. In other parts of the world, the medicinal use of bryophytes is rather sporadic.

The first reports on the antimicrobial effects of bryophytes date back to the 1940s. In the 1950s, the remarkable antibacterial effect of some species (*Anomodon rostratus*, *Orthotrichum rupestre*, and *Plagiomnium cuspidatum*) [8] attracted scientific interest, and some years later, the first extensive study on this topic, including the analysis of 50 species, was published [9]. In a comprehensive study published in 1979, the antibiotic activity of 52 species was examined on 8 bacterial strains; 56% of the tested species were active against at least one of the test bacteria [10]. Since then, several bryophytes, including the most active from the *Bazzania*, *Conocephalum*, *Diplophyllum*, *Dumortiera*, *Marchantia*, *Metzgeria*, *Lunularia*, *Pellia*, *Plagiochila*, *Porella*, *Radula*, and *Riccardia* genera, were reported to have antimicrobial activity [11,12]. In liverworts, essential oils may partly be responsible for this activity [13]; however, in several cases, active components have been identified from the involatile fraction. Sacculatal from *Pellia endiviifolia* showed potent antibacterial activity against *Streptococcus mutans* [14], lepidozenolide from *Lepidozia fauriana* showed activity against methicillin-resistant *Staphylococcus aureus* (MRSA) [15], and marchantins from many *Marchantia* species showed activity against more than 10 pathogen bacteria [16,17]. Herbertane sesquiterpenoids were active against *Staphylococcus aureus*, *Klebisella pneumoniae*, and *Bacillus subtilis* [18,19]. Chlorinated bibenzyls from *Riccardia marginata* exhibited antibacterial effect against *Bacillus subtilis* [12]. A series of diterpenoids from the liverwort *Jungermannia exertifolia* showed potent activity against the virulent *Mycobacterium tuberculosis* H37Rυ strain [20]. 

Extracts of several bryophytes and certain secondary metabolites exhibited remarkable in vitro cytotoxicity on cancer cell lines. Terpenoids and bibenzyl derivatives have been reported among the most potent cytotoxic compounds [21]. Sesquiterpenoids (among them, lactones), such as plagiochiline A and its derivatives [16,22], costunolide, and related compounds [23], exhibited cytotoxicity at micromolar and submicromolar concentrations. Certain diterpenes, including kaurane (e.g., rabdoumbrosanin and derivatives) [24,25], pimarane [11], and atisane-type compounds [26], were also confirmed to be active. Macrocyclic bis(bibenzyls) such as marchantins and riccardins are characteristic compounds of bryophytes. Their *in vitro* antiproliferative activities have been confirmed in several experiments [17,27]. The latter groups of compounds—similarly to certain kaurane diterpenes [28]—exert their effects through induction of apoptosis [29,30] and inhibit *p*-glycoprotein-mediated multidrug resistance [31]. Some ent-kauranes induced apoptosis through a caspase-8-dependent pathway [32,33]. In the case of diterpene jungermanenones, a caspase-independent pathway, together with the inhibition of NF-κB (nuclear factor kappa-light-chain-enhancer of activated B cells), is suggested [34]. Marchantin C, a compound with a different structure from all the previously known microtubule inhibitors, decreased the quantity of microtubules at the G2/M phase in human tumor cells and decreased the polymerisation rate of tubulin, similarly to in [30,35].

The aim of our work was to carry out an extensive *in vitro* bioactivity assay on 42 bryophyte species native to Hungary to identify taxa with remarkable antiproliferative and antimicrobial activities. Further, the phytochemical and bioactivity data of the assayed species were reviewed.

## 2. Results

In the course of the in vitro screening for antiproliferative and antimicrobial activities, 168 extracts belonging to 42 bryophyte species, 35 genera, and 20 families were evaluated using the 3-(4,5-dimethylthiazol-2-yl)-2,5-diphenyltetrazolium bromide (MTT) assay and the disc-diffusion method, respectively. The antiproliferative activities of the extracts were assessed on human cervical (HeLa), ovarian (A2780), and breast (T47D) cancer cell lines using two final concentrations (10 and 30 µg/mL). The results of the antiproliferative assays are presented in Table 1. Extracts prepared with *n*-hexane (A), CHCl_3_ (B), aqueous MeOH (C), and H_2_O (D) were investigated for their cytostatic effects. A total of 98 extracts representing 41 species exerted ≥25% inhibition of proliferation of at least one of the cell lines at 10 μg/mL. The numbers of active A/B/C/D fractions were 24/38/20/16, respectively. In the case of 25 extracts (from 17 species), the inhibition was ≥50% on at least one of the cell lines at this concentration. This higher inhibition was most characteristic to B extracts (13), followed by A (7), C (4), and D (1). More than one extract was active in the case of six species, namely, *Brachythecium rutabulum*, *Climacium dendroides*, *Encalypta streptocarpa*, *Pleurozium schreberi* (A and B), *Neckera besseri*, and *Pseudoleskeella nervosa* (A, B, and C). At 30 μg/mL, 36 samples belonging to 26 species were inactive (25 of these were D extracts); further analysis of these extracts did not seem to be prospective. Compounds responsible for bioactivity were less polar in the analysed samples. The highest activity (78.54% inhibition on HeLa at 10 μg/mL) was observed in the case of the B extract of *Paraleucobryum longifolium*. Moreover, this extract was active on all the cell lines, and activities at 10 μg/mL were not much lower than those at 30 μg/mL (46.84–78.54% vs 56.87–83.93%). Interestingly, in the case of this species, only the CHCl_3_ extract had remarkable activities. Concerning the sensitivity of the cell lines, the measure of inhibition was more pronounced in the cases of HeLa and T47D than A2780. On HeLa, 16 extracts; on T47D, 10 extracts; and on A2780, only 3 extracts exerted >50% inhibition at 10 μg/mL. From the tested families, Brachytheciaceae (with *Brachythecium rutabulum*, *Homalothecium philippeanum*, and *Pseudoscleropodium purum*) and Amblystegiaceae (with *Amblystegium serpens* and *Hygroamblystegium tenax*) provided the highest numbers of active extracts.

The antimicrobial activity of the tested bryophytes, determined by the disc-diffusion method on 11 standard strains, seemed to be sporadic and of low intensity. From the 42 tested species, only 19 samples of 15 taxa showed moderate antibacterial activity (Table 2). None of the extracts were active on *Pseudomonas aeruginosa* ATCC 27853, *Escherichia coli* ATCC 35218, and *Klebsiella pneumoniae* ATCC 700603. Methicillin-resistant *Staphylococcus aureus* ATCC 43300 and *Staphylococcus aureus* ATCC 29213 proved to be the strains most susceptible to the examined species. Among the fractions with different polarities, the relatively apolar *n*-hexane and chloroform extracts demonstrated antibacterial activities. The aqueous and remnant MeOH fractions were inactive. From the tested species, *Plagiomnium cuspidatum* seemed to be the most prospective for further analysis of its antibacterial effects and constituents responsible for this activity, being active on eight tested strains.

There was a notable correlation between the antiproliferative and antimicrobial activities: 7 of the 14 species with antimicrobial activities and 7 of the 15 species with >50% inhibitory activity at 10 μg/mL on any of the cancer cell lines also possessed the other activity. *Amblystegium serpens*, *Brachythecium rutabulum*, *Cirriphyllum piliferum*, *Climacium dendroides*, *Paraleucobryum longifolium*, *Plagiomnium affine*, and *Pseudoscleropodium purum* were active in both assays. 

For 14 species active in either of the assays, no chemical or pharmacological data are available in the literature (*Amblystegium serpens* (Hedw.) Schimp., *Barbula unguiculata* Hedw., *Bryum caespiticium* Hedw., *Cirriphyllum piliferum* (Hedw.) Grout, *Dicranum tauricum* Sapjegin, *Encalypta streptocarpa* Hedw., *Hygroamblystegium tenax* (Hedw.) Jenn., *Neckera besseri* (Lobarz.) Jur., *Oxyrrhyncium hians* (Hedw.) Loeske, *Paraleucobryum longifolium* (Hedw.) Loeske, *Pseudoleskeella nervosa* (Brid.) Nyholm, *Schistidium crassipilum* H. H. Blom, *Syntrichia ruralis* (Hedw.) F. Weber & D. Mohr, and *Thuidium assimile* (Mitt.) A. Jaeger) (Table 3). Ethnopharmacological data are scarce. *Barbula unguiculata* has been used as an analgesic and to reduce fever, and *Bryum argenteum* has been used as an antipyretic and as an antifungal agent in folk medicine [36]. The available data on biologically active species generally do not confirm or support our observations. In *Anomodon viticulosus*, previously only fatty acids were detected [37,38]; there are no data in literature on its antiproliferative and antimicrobial effects. In the case of *Atrichum undulatum*, the presence of the reported sterols [38], carotenoids [38], and fatty acids [39] may not be related to the antiproliferative activity; however, coumarin glycosides [40] may have some role in this effect. The DMSO (dimethyl sulfoxide) and aqueous extracts of this species were active against certain bacteria [41,42]; however the extracts analysed by us did not have such an effect. For *Brachytheciastrum velutinum*, only the presence of phenolic acids and flavonoids has been reported [43]. *Brachythecium rutabulum* possessed remarkable antibacterial activity [44], including against *Staphylococcus aureus*, as also demonstrated in our experiments. *Bryum argenteum*, with a confirmed flavonoid content [45,46], was reported to be active against different bacterial and fungal strains [8,47,48]. However, in our antimicrobial assay, it was not active. For *Calliergonella cuspidata*, only the presence of fatty acids [49] and antioxidant activity was reported [50], with no reference to the antimicrobial activity first observed by us. In the case of *Climacium dendroides*, both antiproliferative and antimicrobial effects have been reported [51]. From the secondary metabolites of this species, flavonoids and chromenones [51,52] might be related with these activities; however, the role of these compounds has not been investigated. *Homalothecium philippeanum* was antiproliferative in our assay but, contrary to previous results [53], exerted no antibacterial activity against *Staphylococcus aureus*. From *Plagiomnium affine*, which was active in both of our assays, only the presence of flavonoids has been reported previously [54]. Two other *Plagiomnium* species (*P. cuspidatum* and *P. undulatum*) had antimicrobial activities in our tests, with literature references only to their flavonoid contents [55,56,57,58]. In contrast to previous reports [53,59], *Pleurozium schreberi* did not exert antimicrobial activity*.* The sesquiterpene and flavonoid content of *Porella platyphylla* [60,61,62,63] may be related to its antiproliferative activity, but there are no reports on the anticancer effect of this species. From *Pseudoscleropodium purum*, only sterols, triterpenes, and essential oil have been reported [64,65,66]. The antimicrobial activity of *Rhytidium rugosum* was reported previously [53]. As part of our experiments, we carried out an LC-MS-based characterisation of the most biologically active extracts. Altogether, 58 compounds were identified from 9 different extracts (Table S4), the majority of which were acids and terpenoids. The limitation of this approach was that it allowed the identification of already known compounds. Further experiments will aim at the isolation and identification of bioactive constituents.

## 3. Discussion

The present investigation aimed at screening for antiproliferative and antimicrobial activities of selected bryophytes collected in Hungary. Our results and the lack of extensive scientific data on biologically active species suggest the necessity of further phytochemical and biological investigations. For the 14 species having antiproliferative or antimicrobial effects, this is the first report on their bioactivities.

## 4. Materials and Methods 

### 4.1. Plant Material

Bryophytes were collected in the Northern Medium Mountains (Hungary) in September and October of 2014 and were identified by Péter Szűcs. Voucher specimens for each plant were deposited at the herbarium of the Institute of Pharmacognosy, University of Szeged. Extracts were prepared according to the method described previously [91]. Briefly, air-dried, powdered plant material was extracted using MeOH with the use of an ultrasonic bath. After filtration and evaporation, the residues were dissolved in 50% aqueous MeOH and subjected to solvent–solvent partition between *n*-hexane (extracts A) and CHCl_3_ (extracts B), and the remnant gave extracts C. The residual plant materials were dried and extracted with boiling H_2_O. The filtered extracts were freeze-dried, affording extracts D.

### 4.2. Antiproliferative Assay

The antiproliferative properties of the prepared extracts were determined by means of the MTT assay on a panel of human adherent cancerous cell lines of gynaecological origin containing A2780, HeLa, and T47D cells isolated from ovarian, cervical, and breast carcinomas, respectively. All the cells were purchased from ECACC (European Collection of Authenticated Cell Cultures, Salisbury, U.K.) and were cultivated in minimal essential medium supplemented with 10% fetal bovine serum, 1% non-essential amino acids, and an antibiotic–antimycotic mixture. All media and supplements were obtained from Lonza Group Ltd. (Basel, Switzerland). The cells were maintained at 37 °C in humidified atmosphere containing 5% CO_2_. Near-confluent cancer cells were seeded onto a 96-well microplate (5000 per well) and attached to the bottom of the well overnight. On the second day, 200 μL of new medium containing the tested substances (at 10 or 30 µg/mL) was added. After incubation for 72 h, the living cells were assayed by the addition of 20 μL of 5 mg/mL MTT (3-(4,5-dimethylthiazol-2-yl)-2,5-diphenyltetrazolium bromide) solution. MTT was converted by intact mitochondrial reductase and precipitated as blue crystals during a 4 h contact period. The medium was then removed, and the precipitated crystals were dissolved in 100 μL of DMSO during a 60 min period of shaking at 25 °C. Finally, the reduced MTT was assayed at 545 nm using a microplate reader; wells with untreated cells were used as controls [92]. All experiments were carried out on two microplates with at least five parallel wells. Stock solutions of the tested substances (10 mg/mL) were prepared with DMSO. The highest DMSO content of the medium (0.3%) did not have any substantial effect on the cell proliferation. Cisplatin (Ebewe Pharma GmbH, Unterach, Austria), a clinically utilised anticancer drug, was used as a reference agent. The IC_50_ values of its antiproliferative action were 12.43, 1.30, and 9.78 μM against HeLa, A2780, and T47D cells, respectively. 

### 4.3. Antimicrobial Assay

Antibacterial activities of the extracts against standard bacterial strains were screened for their inhibition zones by the standard disc-diffusion method described previously [93]. The test microorganisms used in this study were 11 international control standard strains. The standard Gram-positive strains were, namely, *Bacillus subtilis* (ATCC 6633), *Staphylococcus aureus* (ATCC 29213), *Staphylococcus epidermidis* (ATCC 12228), *Streptococcus agalactiae* (ATCC 13813), *Streptococcus pneumoniae* (ATCC 49619), *Streptococcus pyogenes* (ATCC 19615), and methicillin-resistant *Staphylococcus aureus* (ATCC 43300). The standard Gram-negative strains were, namely, *Escherichia coli* (ATCC 35218), *Klebsiella pneumoniae* (ATCC 700603), and *Moraxella catarrhalis* (ATCC 43617). Microbial cultures were grown on standard Mueller-Hinton agar plates or Columbia agar +5% sheep blood (COS) plates (bioMérieux, Marcy-l’Étoile, France) at 37 °C under an aerobic or 5% CO_2_ environment. The strains were stored in Cryobank vials (MAST Diagnostica, Rheinfeld, Germany) at −70 °C and maintained at 4 °C throughout the study to use as stock cultures. Briefly, bryophyte extracts were dissolved in DMSO (Sigma-Aldrich, St. Louis, MO, USA) or water at a concentration of 50 mg mL^−1^. The sterile filter paper discs (6 mm in diameter) impregnated with the extracts (10 uL of redissolved extracts) were placed on the plate seeded with the respective bacterial suspensions (inoculums: 0.5 McFarland, 1–2 × 10^8^ CFU mL^−1^). The solvent (DMSO) served as the negative control, while ampicillin, erythromycin, imipenem, cefuroxime, and vancomycin antibiotic susceptibility discs were used as the positive control. The plates were incubated at 37 °C for 24 h under aerobic or 5% CO_2_ conditions. The diameters of inhibition zones produced by the extracts (including the disc) were measured and recorded. All experiments were carried out in triplicate.

### 4.4. Phytochemical Characterisation of the Extracts

The most active samples were analysed phytochemically by LC-MS. Samples were filtered through 0.45 µm PTFE (polytetrafluoroethylene) syringe filters before analysis. For separation, Kinetex (Phenomenex; XB-C18 and Phenyl-Hexyl, 2.1 × 100 mm, 2.6 um, 100 Å) columns were used. The eluents were the following: A: 0.1% formic acid in MS-grade water; B: 0.1% formic acid in MS-grade acetonitrile. The separation was done with a linear gradient from 5% to 95% B in 35 min with a 0.3 mL/min flow rate. The HPLC instrument was an Agilent 1100 series model consisting of a binary pump, a thermostated autosampler, and a column compartment. The mass spectrometer was a Thermo Q-Exactive Plus Orbitrap equipped with a HESI-II ion source. The mass accuracy was 0.25+/−0.01 ppm in the positive mode and 0.34+/−0.05 in the negative mode at the mass calibration just before the experiment. Acquisition was done in the data-dependent MS^2^ scan mode by altering the charge state (positive/negative). The survey scan mass range was set to *m*/*z* 80–1000, using the lock masses from the known background ions listed in Table S2. The data-dependent method parameters are shown in Table S3. The acquired MS^2^ peak lists were converted to a text file by using the msConvert tool (Proteowizard), and the top 100 MS survey scan peaks were chosen for MS^2^ identification against KEGG’s small-molecule database, using the MetFrag online search tool. The hits were filtered manually using an 80% matched peak result when the number of MS^2^ fragment peaks was at least five.

## Figures and Tables

**Table 1:** Antiproliferative activities against cancer cell lines treated with extracts for an exposure time of 72 h; values exceeding 50% inhibition are coloured from yellow to green; extracts exerting less than 25% inhibition of cancer cell growth were considered inactive (red), and their exact results are not given numerically; SEM (standard error of the mean) values reported in Table S1.

**Species**	**Extract A**						**Extract B**					
	**HeLa**		**A2780**		**T47D**		**HeLa**		**A2780**		**T47D**	
	**10 µg/mL**	**30 µg/mL**	**10 µg/mL**	**30 µg/mL**	**10 µg/mL**	**30 µg/mL**	**10 µg/mL**	**30 µg/mL**	**10 µg/mL**	**30 µg/mL**	**10 µg/mL**	**30 µg/mL**
*Abietinella abietina*	<25	<25	<25	<25	<25	32.26	30.00	38.62	<25	<25	42.99	48.97
*Amblystegium serpens*	<25	46.13	29.58	49.94	49.15	70.15	61.93	70.78	53.46	65.35	70.15	74.76
*Anomodon viticulosus*	26.96	50.72	<25	<25	<25	27.81	27.04	49.35	32.35	53.87	<25	36.32
*Atrichum undulatum*	<25	<25	<25	<25	<25	41.66	59.93	76.26	37.78	64.28	64.11	65.26
*Barbula unguiculata*	45.74	63.27	<25	<25	<25	34.14	65.46	75.11	<25	47.47	44.20	53.16
*Brachytheciastrum velutinum*	31.92	64.96	<25	35.58	<25	41.01	34.43	55.09	<25	61.29	34.43	51.51
*Brachythecium rutabulum*	53.49	61.64	25.04	34.93	45.40	55.36	51.95	53.89	<25	35.30	46.79	54.92
*Bryum argenteum*	47.79	80.09	<25	<25	<25	<25	36.11	54.52	<25	<25	35.95	41.26
*Bryum caespiticium*	30.37	57.84	<25	<25	<25	48.80	48.64	59.57	<25	<25	28.58	48.17
*Bryum moravicum*	<25	38.27	<25	<25	<25	29.73	46.72	62.09	27.34	48.22	40.64	59.69
*Calliergonella cuspidata*	<25	<25	<25	<25	<25	<25	<25	32.79	<25	<25	39.02	49.49
*Ceratodon purpureus*	<25	26.67	<25	32.49	<25	28.88	30.67	42.00	<25	35.18	<25	28.48
*Cirriphyllum piliferum*	51.34	67.39	<25	42.24	<25	31.19	<25	28.32	<25	<25	<25	<25
*Climacium dendroides*	52.79	63.79	<25	32.63	<25	<25	56.79	64.89	<25	<25	55.46	57.16
*Dicranum tauricum*	<25	<25	<25	28.55	<25	31.60	33.14	51.60	<25	48.94	35.38	54.93
*Encalypta streptocarpa*	76.66	61.32	34.04	87.90	25.72	44.08	54.46	72.90	73.72	80.12	33.22	33.27
*Funaria hygrometrica*	<25	39.84	<25	<25	<25	36.21	48.44	62.88	25.66	51.06	46.44	53.18
*Homalothecium lutescens*	<25	37.79	<25	31.59	<25	30.29	<25	<25	<25	<25	28.74	30.64
*Homalothecium philippeanum*	38.34	63.66	<25	40.77	37.39	48.19	46.93	73.77	33.60	74.93	63.90	62.51
*Hygroamblystegium tenax*	<25	51.08	<25	26.53	28.34	43.75	36.99	43.86	<25	<25	49.19	55.28
*Leskea polycarpa*	<25	29.00	<25	37.18	<25	25.71	<25	31.32	<25	35.02	<25	37.07
*Leucodon sciuroides*	26.00	43.88	<25	<25	<25	34.43	42.48	61.74	<25	<25	28.88	39.63
*Neckera besseri*	54.29	68.98	<25	<25	33.72	38.33	69.13	83.28	<25	76.48	50.07	68.26
*Orthotrichum diaphanum*	<25	28.22	<25	<25	<25	<25	40.19	51.75	25.04	50.65	35.43	40.61
*Oxyrrhynchium hians*	<25	50.41	<25	42.22	26.03	46.46	25.65	39.79	<25	28.61	34.01	46.64
*Paraleucobryum longifolium*	<25	27.34	<25	<25	<25	<25	78.54	83.93	63.23	78.03	46.84	56.87
*Plagiomnium affine*	<25	41.79	<25	<25	<25	<25	42.41	55.53	<25	42.11	42.49	56.05
*Plagiomnium cuspidatum*	39.13	39.44	26.49	97.60	<25	86.33	<25	<25	<25	56.15	<25	36.11
*Plagiomnium rostratum*	26.01	60.72	<25	44.99	28.68	36.26	46.52	60.22	43.23	67.06	45.56	54.59
*Plagiomnium undulatum*	35.77	33.42	<25	26.07	29.36	43.49	<25	<25	<25	33.21	<25	32.10
*Pleurozium schreberi*	61.85	93.41	41.15	37.25	<25	31.65	60.49	74.30	<25	36.89	29.26	43.95
*Pohlia nutans*	<25	<25	<25	<25	<25	<25	29.51	49.60	<25	<25	<25	<25
*Polytrichastrum formosum*	<25	34.69	<25	<25	<25	28.85	<25	34.18	<25	<25	<25	<25
*Porella platyphylla*	31.89	79.22	48.22	83.33	48.94	64.37	35.69	47.36	<25	41.93	29.33	47.86
*Pseudoleskeella nervosa*	68.43	75.64	<25	<25	38.94	43.69	61.71	71.88	<25	36.28	42.77	45.50
*Pseudoscleropodium purum*	<25	34.16	<25	<25	<25	<25	62.06	70.27	<25	28.01	53.88	54.58
*Rhytidiadelphus squarrosus*	<25	<25	<25	<25	<25	<25	43.99	53.66	<25	<25	40.31	51.65
*Rhytidium rugosum*	32.34	56.50	<25	36.29	<25	25.80	30.20	39.48	<25	<25	<25	27.52
*Schistidium crassipilum*	<25	33.32	<25	<25	<25	<25	27.52	53.09	<25	72.36	<25	38.36
*Syntrichia ruralis*	<25	<25	<25	<25	<25	<25	27.05	33.02	<25	<25	30.49	39.25
*Thamnobryum alopecurum*	29.98	57.12	<25	26.75	<25	<25	34.35	53.87	<25	51.91	<25	<25
*Thuidium assimile*	<25	<25	<25	29.18	<25	<25	43.36	57.09	34.62	58.86	65.70	56.12
**Species**	**Extract C**						**Extract D**					
	**HeLa**		**A2780**		**T47D**		**HeLa**		**A2780**		**T47D**	
	**10 µg/mL**	**30 µg/mL**	**10 µg/mL**	**30 µg/mL**	**10 µg/mL**	**30 µg/mL**	**10 µg/mL**	**30 µg/mL**	**10 µg/mL**	**30 µg/mL**	**10 µg/mL**	**30 µg/mL**
*Abietinella abietina*	35.25	52.48	<25	<25	38.22	49.01	<25	<25	<25	<25	<25	30.04
*Amblystegium serpens*	33.19	44.83	<25	26.71	48.58	58.34	<25	<25	<25	<25	27.91	35.73
*Anomodon viticulosus*	<25	34.02	<25	<25	<25	27.47	<25	<25	<25	<25	<25	<25
*Atrichum undulatum*	29.14	41.65	<25	46.14	<25	37.95	33.37	36.14	<25	<25	<25	<25
*Barbula unguiculata*	29.68	35.91	<25	<25	<25	27.68	27.92	27.4	<25	<25	<25	<25
*Brachytheciastrum velutinum*	<25	32.09	<25	<25	27.26	34.7	34.9	34.68	<25	<25	38.53	38.46
*Brachythecium rutabulum*	<25	34.26	<25	<25	<25	34.81	<25	<25	<25	<25	<25	<25
*Bryum argenteum*	<25	<25	<25	<25	<25	<25	<25	<25	<25	<25	<25	<25
*Bryum caespiticium*	<25	35.57	<25	<25	<25	<25	<25	<25	<25	<25	<25	<25
*Bryum moravicum*	<25	37.94	<25	<25	<25	26.51	<25	<25	<25	<25	<25	<25
*Calliergonella cuspidata*	<25	<25	<25	<25	<25	<25	<25	<25	<25	<25	<25	<25
*Ceratodon purpureus*	<25	<25	<25	<25	<25	31.86	<25	<25	<25	<25	<25	<25
*Cirriphyllum piliferum*	28.18	42.07	<25	<25	<25	26.85	<25	32.27	<25	<25	<25	<25
*Climacium dendroides*	<25	<25	<25	<25	<25	27.68	<25	27.42	<25	<25	27.38	37.52
*Dicranum tauricum*	<25	28.29	<25	<25	33.52	49.97	29.75	37.11	<25	<25	45.31	47.21
*Encalypta streptocarpa*	28.01	39.61	<25	<25	<25	32.5	27.05	<25	<25	<25	<25	<25
*Funaria hygrometrica*	<25	<25	<25	<25	42.27	48.22	25.11	38.47	<25	<25	35.4	45.16
*Homalothecium lutescens*	<25	<25	<25	<25	<25	<25	<25	<25	<25	<25	<25	27.6
*Homalothecium philippeanum*	<25	33.51	<25	28.04	43.32	51	<25	<25	<25	<25	33.68	41.04
*Hygroamblystegium tenax*	26.66	38.22	<25	<25	52.69	55.03	<25	31.71	<25	<25	37.34	40.38
*Leskea polycarpa*	25.62	31.09	<25	<25	<25	34.12	<25	<25	<25	<25	<25	<25
*Leucodon sciuroides*	<25	29.98	<25	<25	<25	<25	<25	<25	<25	<25	<25	<25
*Neckera besseri*	37.13	41.25	<25	<25	54.5	55.63	<25	<25	<25	<25	32.28	43.28
*Orthotrichum diaphanum*	<25	40.79	<25	<25	<25	28.2	<25	<25	<25	<25	<25	<25
*Oxyrrhynchium hians*	<25	<25	<25	<25	<25	29.53	<25	<25	<25	<25	<25	<25
*Paraleucobryum longifolium*	<25	<25	<25	<25	<25	<25	<25	<25	<25	<25	<25	<25
*Plagiomnium affine*	42.04	50.67	<25	26.86	53.3	57.53	<25	<25	<25	<25	<25	<25
*Plagiomnium cuspidatum*	35.49	46.35	<25	<25	33.53	45.9	<25	<25	<25	<25	<25	27.79
*Plagiomnium rostratum*	40.44	51.65	<25	42.42	33.23	45.84	<25	<25	<25	<25	<25	<25
*Plagiomnium undulatum*	<25	<25	<25	<25	<25	<25	<25	<25	<25	<25	<25	<25
*Pleurozium schreberi*	<25	32.99	<25	<25	<25	<25	<25	<25	<25	<25	<25	<25
*Pohlia nutans*	<25	32.63	<25	<25	<25	<25	<25	<25	<25	<25	<25	<25
*Polytrichastrum formosum*	<25	<25	<25	<25	<25	<25	<25	<25	<25	<25	<25	<25
*Porella platyphylla*	<25	41.69	<25	<25	<25	27.66	<25	<25	<25	<25	<25	<25
*Pseudoleskeella nervosa*	60.51	65.03	<25	26.27	49.89	54.5	<25	<25	<25	<25	<25	25.43
*Pseudoscleropodium purum*	<25	28.06	<25	<25	<25	31.29	<25	29.89	<25	<25	32.22	40.05
*Rhytidiadelphus squarrosus*	<25	26.56	<25	<25	<25	33.78	<25	<25	<25	<25	<25	<25
*Rhytidium rugosum*	<25	33.67	<25	<25	<25	<25	<25	<25	<25	<25	<25	<25
*Schistidium crassipilum*	<25	<25	<25	<25	<25	<25	<25	<25	<25	<25	<25	<25
*Syntrichia ruralis*	<25	<25	<25	<25	<25	<25	30.83	41.17	<25	<25	57.42	59.35
*Thamnobryum alopecurum*	<25	31.9	<25	<25	<25	<25	26.26	40.68	<25	<25	<25	<25
*Thuidium assimile*	<25	<25	<25	34.23	29.78	43.24	<25	<25	<25	<25	32.28	44.58

**Table 2:** Antibacterial activities of moss extracts (inhibition zones in millimetres).

Species	Extract	MRSA	*S. aureus*	*S. epidermidis*	*B. subtilis*	*S. pyogenes*	*S. pneumoniae*	*S. agalactiae*	*M. catarrhalis*
		ATCC 43300	ATCC 29213	ATCC 12228	ATCC 6633	ATCC 19615	ATCC 49619	ATCC 13813	ATCC 43617
*Amblystegium serpens*	B	—	—	—	—	—	—	—	9.0
*Brachythecium rutabulum*	B	9.0	9.0	—	—	—	—	—	—
*Calliergonella cuspidata*	A	—	7.3	—	—	—	—	—	—
	B	—	7.0	—	—	—	—	—	—
*Cirriphyllum piliferum*	B	—	—	—	—	—	7.0	—	—
*Climacium dendroides*	A	—	7.3	—	—	—	—	—	—
*Dicranum tauricum*	B	—	—	—	—	—	8.0	—	—
*Oxyrrhynchium hians*	A	8.6	8.6	—	—	—	—	—	—
	B	—	8.0	—	—	—	—	—	—
*Paraleucobryum longifolium*	B	9.6	9.6	—	—	11.6	—	—	—
*Plagiomnium affine*	B	—	—	—	8.0	—	8.5	—	—
*Plagiomnium cuspidatum*	A	11.3	10.7	9.0	9.0	10.0	12.0	10.0	10.0
	B	7.6	7.6	—	—	—	—	—	—
*Plagiomnium undulatum*	A	7.0	8.0	—	—	—	—	—	—
	B	—	8.0	—	—	—	—	—	—
*Pseudoscleropodium purum*	A	—	7.3	—	—	—	—	—	—
*Rhytidium rugosum*	B	—	—	—	7.5	—	8.0	—	7.5
*Schistidium crassipilum*	B	8.0	7.0	—	9.0	—	11.5	—	7.7

**Table 3:** Literature data on the chemistry (C) and bioactivities (B) of the studied species.

Species (Family)	Bioactivity	
*Abietinella abietina* (Hedw.) M. Fleisch. (Thuidiaceae)	C	Fatty acids (main components: oleic, palmitic, and linoleic acid) [67]; sterols (sitosterol, stigmasterol, and campesterol) [67,68]
	B	Antimicrobial effect with MIC (minimum inhibitory concentration) values of 1.25–10 mg/mL against Gram-positive (*Staphylococcus aureus*, *Micrococcus flavus*, and *Bacillus cereus*) and Gram-negative (*Escherichia coli* and *Salmonella typhimurium*) bacteria and fungi (*Trichoderma viride*, *Penicillium funiculosum*, *Penicillium ochrochloron*, *Aspergillus flavus*, *A. niger*, *and A. fumigatus*) [69]
*Anomodon viticulosus* (Hedw.) Hook. & Taylor (Thuidiaceae)	C	Fatty acids (linoleic acid, nonadecanoic acid, palmitic acid, and behenic acid) [37,38]
*Atrichum undulatum* (Hedw.) P. Beauv. (Polytrichaceae)	C	Sterols (major: 24-methylcholesterol and 24-ethyl-22-dehydrocholesterol) [38]; carotenoids (β-carotene, lutein, violaxanthin, and neoxanthin) [38]; fatty acids (major: linoleic acid, α -linolenic acid, palmitic acid, oleic acid, and arachidonic acid) [39]; coumarin glycosides [40]
	B	Antimicrobial effect of DMSO extracts with MIC values of 0.5–3.0 mg/mL against eight bacterial species (*Escherichia coli*, *Pseudomonas aeruginosa*, *Salmonella typhimurium*, *Enterobacter cloacae*, *Listeria monocytogenes*, *Bacillus cereus*, *Micrococcus flavus*, and *Staphylococcus aureus*) [41]; aqueous extract active against *Staphylococcus aureus* [42]; weak antioxidant activity in vitro [70]
*Brachythecium rutabulum* (Hedw.) Schimp. (Brachytheciaceae)	C	Polyunsaturated fatty acids [71]
	B	The EtOH extract active against the bacteria *Micrococcus luteus*, *Bacillus subtilis*, *Bacillus cereus*, *Enterobacter aerogenes*, *Escherichia coli*, *Klebsiella pneumoniae*, *Proteus mirabilis*, *Pseudomonas aeruginosa*, *Staphylococcus aureus*, *Salmonella typhimurium*, and *Streptococcus pneumoniae* and the fungi *Candida albicans*, *Cryptococcus albidus*, *Trichophyton rubrum*, *Aspergillus niger*, and *Aspergillus flavus* with MIC values of 0.19–1.56 μg/mL [44]; antioxidant activity [50]
*Brachytheciastrum velutinum* (Hedw.) Ignatov & Huttunen (Brachytheciaceae)	C	Phenolic acids (4-*O*-caffeoylquinic, 5-*O*-caffeoylquinic, and caffeic and ellagic acids) and flavonoids (apigenin-7-*O*-glucoside, luteolin, and apigenin) [43]
*Bryum argenteum* Hedw. (Bryaceae)	C	Major flavonoid glycosides in Antarctic *B. argenteum* samples apigenin and luteolin glucosides and their 6″-malonyl esters, and the 7-O-glucosides of 8-hydroxyapigenin and 8-hydroxyluteolin [45]; luteolin and apigenin content between 0.1 and 0.6 mg/g [46]
	B	In vitro antimicrobial effects of different extracts against *Bacillus cereus*, *Escherichia coli*, *Klebsiella pneumoniae*, *Pseudomonas aeruginosa*, *Staphylococcus aureus*, *Enterococcus faecalis*, *Enterobacter aerogenes*, and *Proteus mirabilis*. Highest activity against *E. coli* and *S. aureus* (MICs of 30–70 μg/mL) [47]; no activity of different extracts against *Staphylococcus aureus*, *Salmonella pullorum*, *Phytomonas phaseoli*, *Candida albicans*, *Salmonella paratyphi*, *Micrococcus flavus*, *Shigella flexneri*, *Micrococcus rubens,* or *Streptococcus pyogenes* [8]; an EtOH extract exerted antimicrobial activity on *Escherichia coli*, *Bacillus subtilis*, *Micrococcus luteus*, *Staphylococcus aureus*, *Aspergillus niger*, *Penicilliumo chrochloron*, *Candida albicans*, and *Trichophyton mentagrophyes* with MICs of 0.10–0.41 mg/mL [48]
*Bryum moravicum* Podp. (Bryaceae)	C	High α-linolenic acid content [72]
	B	Moderate antioxidant activity in vitro [73]
*Calliergonella cuspidata* (Hedw.) Loeske (Hypnaceae)	C	Fatty acids (major: palmitic acid, stearic acid, oleic acid, and linolenic acid [49]
	B	Weak antioxidant activity in vitro [50]
*Ceratodon purpureus* (Hedw.) Brid. (Ditrichaceae)	C	Flavonoid (lutelolin) [74]; polyacetylenes [75]; fatty acids (Ω-3 and -6) [38]; five new isopimarane diterpenes—smardaesidins A–E—and two new 20-nor-isopimarane diterpenes—smardaesidins F and G—together with sphaeropsidins A and C–F were isolated from an endophytic fungal strain, *Smardaea* sp. AZ0432, obtained from *Ceratodon purpureus* [76]
	B	A MeOH extract with moderate effect against methicillin-resistant *Staphylococcus aureus* [59]; moderate antioxidant activity of the EtOH extract, independent from the total phenolic content [77]; sphaeropsidin A and D cytotoxic on different cancer cell lines, and sphaeropsidin A inhibited the migration of metastatic breast adenocarcinoma (MDA-MB-231) cells at subcytotoxic concentration [76]
*Climacium dendroides* (Hedw.) F. Weber & D. Mohr(Climaciaceae)	C	Sterols (major: sitosterol, stigmasterol, and campesterol) [67]; organic acids and flavonoid (apigenin) [51]; chromenone derivatives and flavonoids (kaempferol and quercetin glycosides) [52]
	B	EtOH extract with weak antiproliferative effect on different animal and human cancer cell lines and remarkable antimicrobial activity against *Escherichia coli*, *Bacillus cereus*, and *Staphylococcus aureus* [51]
*Funaria hygrometrica* Hedw. (Funariaceae)	C	Bracteatin, as the first higher plant pigment in mosses, was isolated from this species [78]
	B	Different extracts had weak antimicrobial activities against *Bacillus subtilis*, *Pseudomonas aeruginosa*, and *Staphylococcus aureus* [79]
*Homalothecium lutescens* (Hedw.) H. Rob. (Brachytheciaceae)	C	Flavonoids (3′,3″′-binaringenin and the newly discovered 2,3-dihydro 3′,3″′-biapigenin) [80]
	B	The essential oil was active against the fungi *Candida albicans* and *Saccharomyces cerevisiae* [81]
*Homalothecium philippeanum* (Spruce) Schimp. (Brachytheciaceae)	B	MeOH extract had antibacterial activity against *Staphylococcus aureus*, *Escherichia coli*, *Micrococcus flavus*, and *Salmonella typhimurium* (MICs of 5 mg/mL) and antifungal activity against *Aspergillus niger*, *A. ochraceus*, *A. versicolor*, *Penicillium funiculosum*, *Trichoderma viride*, and *Candida albicans* (MICs of 0.5–2.5 mg/mL) [53]
*Leucodon sciuroides* (Hedw.) Schwägr. (Leucodontaceae)	C	Oxylipins (oct-1-en-3-ol, (*Z*)-octa-1,5-dien-3-ol, (*Z*)-non-2-enal, (*E*)-non-2-enal, (*Z*)-non-3-enal, and 16-(2*E*,6*Z*)-nona-2,6-dienal); essential oil with nonanal and heptanal as main constituents [82]
	B	The MeOH extract had weak to moderate antimicrobial effect against the bacteria *Bacillus subtilis*, *Escherichia coli*, *Klebsiella pneumoniae*, *Pseudomonas aeruginosa*, *Salmonella typhimurium*, *Staphylococcus aureus*, *Streptococcus pyogenes*, and *Mycobacterium smegmatis* and the *fungi Candida albicans*, *Rhodotorula rubra*, *and Kluyveromyces fragilis* [83]; essential oil showed activity against *Candida albicans* [82]
*Plagiomnium affine* (Blandow ex Funck) T. J. Kop. (Mniaceae)	C	Flavonoids (apigenin, vitexin [55], isoorientin, isoorientin 3′-*O*-sophoroside, and isoorientin 3′-*O*-neohesperidoside [54])
*Plagiomnium cuspidatum* (Hedw.) T. J. Kop. (Mniaceae)	C	Flavonoids (saponarine [55]; the 6-*C*-glucosyl-7-*O*-glucosides of apigenin, luteolin, and chrysoeriol; and apigenin-7-*O*-neohesperidoside [56]) and the new dihydrobiflavone 2,3-dihydro-5′-hydroxyamentoflavone, 2,3-dihydro-5′,3′″-dihydroxyamentoflavone, and 2,3-dihydro-5′-hydroxyrobustaflavone [56]
*Plagiomnium undulatum* (Hedw.) T. J. Kop. (Mniaceae)	C	Flavonoids (the biflavonoids 2,3-dihydro-5′-hydroxyrobustaflavone and 2,3-dihydro-5′-hydroxyamentoflavone; the new 3′″-desoxydicranolomin, 2,3-dihydro-3′″-desoxydicranolomin, and 2,3-dihydro-5′,3′″-dihydroxyrobustaflavone [57]; the flavone di-*C*-glycosides schaftoside, isoschaftoside, neoschaftoside, neosisoschaftoside, vicenin-2, and a chrysoeriol 6-C-arabinosyl-8-C-hexoside [58]); essential oil with sesquiterpene hydrocarbons, including γ-elemene as major constituent [84]
*Pleurozium schreberi* (Willd. ex Brid.) Mitt. Hylocomiaceae	C	Apigenin and apigenin-7-rhamnoglucoside [85]
	B	The MeOH extract had weak to moderate activity against *Staphylococcus aureus*, methicilline-resistant *Staphylococcus aureus*, *Bacillus subtilis*, and *Enterococcus faecalis* [59] The MeOH extract was moderately active against *Staphylococcus aureus*, *S. epidermidis*, *Micrococcus flavus*, *Bacillus subtilis*, *Escherichia coli*, *Enterobacter cloacae*, and *Salmonella typhimurium* (MICs of 10-25 mg/mL) and had strong antifungal activity (MIC of 0.5 mg/mL and minimal bactericidal concentration of 2.5–5.0 mg/mL) against *Aspergillus niger*, *A. ochraceus*, *A. versicolor*, *A. flavus*, *Penicillium funiculosum*, *Trichoderma viride*, and *Candida albicans* [53]; the EtOH extract had weak antioxidant activities in different test systems [70]
*Pohlia nutans* (Hedw.) Lindb. (Bryaceae)	C	Essential oil with nonanal and 2*E*-tetradecen-1-ol as major constituents [81]
*Polytrichastrum formosum* (Hedw.) G. L. Sm. (Polytrichaceae)	B	Insecticidal activity of the hexane extract against *Sitophilus granaries* [86]
*Porella platyphylla* (L.) Pfeiff. (Porellaceae)	C	Three new pinguisane-type sesquiterpenes (pinguisanin, pinguisanolide, and β-pinguisenediol) and the previously known deoxopinguisone [60]; a new pinguisanoic acid sesquiterpenoid derivative—methyl 2*a*-hydroxy-6-oxo-11-pinguisanoate—and a new sacculatane diterpenoid hemiacetal—(5*S*,9*S*, 10*R*,13*S*)-ll,13-epoxy-8(12),17-sacculatadiene-13β,15ζ-diol[(13*S*)-15 ζ-hydroxysacculaporellin]—as well as three known pinguisanes (pinguisanin, β-pinguisenediol, and porellapinguisanolide) and the known sacculataneperrottetianal B [61]; flavonoids (isovitexin, saponarin, apigenin-6,8-di-*C*-glycoside [62], schaftoside, vicenin, and isovitexin [63]); perrottetianal B, phytol, and stigmasterol l [87]
	B	Antinociceptive effect of the ether extract (main components: pinguisanin and spiropinguisanine) [88]
*Pseudoscleropodium purum* (Hedw.) M. Fleisch.(Brachytheciaceae)	C	Sterols (24-methyl-5-cholestenol, 24-ethyl-5-cholestenol, and 24-ethyl-5,22-cholestadienol) [64], cyclolaudenol, 31-norcyclolaudenol, campesterol, stigmasterol, and β-sitosterol; the triterpenes hopene, 22(29)-hopene, and ursolic acid [65]; essential oil with the major components α-pinene, β-longipinene, and heptanal [66]
*Rhytidiadelphus squarrosus* (Hedw.) Warnst. (Hylocomiaceae)	C	Sterols (24-methyl-5-cholestenol, 24-ethyl-5-cholestenol, and 24-ethyl-5,22-cholestadienol) [64]; flavonoids (the new biflavone 5′-hydroxyrobusta-flavone and the biflavonoids 5′-hydroxyamentoflavone,5′,3′″-dihydroxyamento-flavone and 2,3-dihydro-5′-hydroxyamentoflavone) [89]
	B	The EtOH extract inhibited the growth of *Staphylococcus aureus* in vitro [89]
*Rhytidium rugosum* (Hedw.) Kindb. (Hylocomiaceae)	C	The major compounds of the essential oil were *n*-hexadecanoic acid, linolenic acid, and *cis*-9- and *cis*-12-octadecadienoic acid. In the diethyl ether extract, ethyl oleate and τ-sitosterol were the most abundant [90]
	B	The MeOH extract exhibited antibacterial effect on *Staphylococcus aureus* and *Micrococcus flavus* (MIC of 5 mg/mL) and antifungal activity against *Aspergillus niger*, *A. ochraceus*, *A. versicolor*, *A. flavus*, *Penicillium funiculosum*, *Trichoderma viride*, and *Candida albicans* with MIC values of 0.5–2.5 mg/mL [53].

## Supplementary Materials

The following are available online: Table S1: Antiproliferative activities against cancer cell lines treated with extracts A–D for an exposure time of 72 h (mean ± SEM); Table S2: The used lock masses and the charge state mode; Table S3: Parameters of the data-dependent acquisition in both charge states; Table S4: Compounds identified by LC-MS.
