# Supplementary material for: Antiproliferative and Antimicrobial Activities of Selected Bryophytes
Source: Molecules. 2018 Jun 23;23(7):1520. doi: 10.3390/molecules23071520 (PMC6099959; doi:10.3390/molecules23071520)
Supplement: Supplementary file 1 [file molecules-23-01520-s001.pdf]

Table S1. Antiproliferative activities against cancer cell lines treated with extracts **A-D** for an exposure time of 72 h (mean ± SEM)

A

Species	HeLa		A2780		T47D	
	10 μg/ml	30 μg/ml	10 μg/ml	30 μg/ml	10 μg/ml	30 μg/ml
Abietinella abietina	<25	<25	<25	<25	<25	32.26 (1.81)
Amblystegium serpens	<25	46.13 (1.05)	29.58 (2.29)	49.94 (1.06)	49.15 (3.12)	70.15 (3.28)
Anomodon viticulosus	26.96 (2.88)	50.72 (4.30)	<25	<25	<25	27.81 (1.62)
Atrichum undulatum	<25	<25	<25	<25	<25	41.66 (2.79)
Barbula unguiculata	45.74 (3.30)	63.27 (3.30)	<25	<25	<25	34.14 (3.67)
Brachytheciastrum velutinum	31.92 (3.13)	64.96 (2.82)	< 25	35.58 (3.26)	< 25	41.01 (2.61)
Brachythecium rutabulum	53.49 (1.88)	61.64 (0.83)	25.04 (1.36)	34.93 (0.74)	45.40 (0.38)	55.36 (1.06)
Bryum argenteum	47.79 (2.39)	80.09 (0.74)	<25	<25	<25	<25
Bryum caespiticium	30.37 (3.18)	57.84 (1.79)	<25	<25	<25	48.89 (2.57)
Bryum moravicum	<25	38.27 (1.49)	<25	<25	<25	29.73 (1.11)
Calliergonella cuspidata	<25	<25	<25	<25	<25	<25
Ceratodon purpureus	<25	26.67 (1.52)	<25	32.49 (4.71)	<25	28.88 (1.13)
Cirriphyllum piliferum	51.34 (3.46)	67.39 (1.85)	<25	42.24 (3.87)	<25	31.19 (4.08)
Climacium dendroides	52.79 (0.91)	63.79 (2.00)	<25	32.63 (0.58)	<25	<25
Dicranum tauricum	<25	<25	<25	28.55 (2.79)	<25	31.60 (1.47)
Encalypta streptocarpa	76.66 (1.91)	61.32 (1.92)	34.04 (3.99)	87.90 (2.05)	25.72 (4.35)	44.08 (1.56)
Funaria hygrometrica	<25	39.84 (1.85)	<25	<25	<25	36.21 (1.25)
Homalothecium lutescens	<25	37.79 (1.04)	<25	31.59 (1.11)	<25	30.29 (1.77)
Homalothecium philippeanum	38.34 (0.98)	63.66 (0.74)	<25	40.77 (1.12)	37.39 (3.01)	48.19 (1.81)
Hygroamblystegium tenax	<25	51.08 (0.38)	<25	26.53 (2.00)	28.34 (2.61)	43.75 (1.75)
Leskea polycarpa	<25	29.00 (1.33)	<25	37.18 (1.37)	<25	25.71 (1.79)
Leucodon sciuroides	26.00 (4.96)	43.88 (2.31)	<25	<25	<25	34.43 (4.20)
Neckera besseri	54.29 (1.27)	68.98 (0.88)	<25	<25	33.72 (1.51)	38.33 (0.96)
Orthotrichum diaphanum	<25	28.22 (1.98)	<25	<25	<25	<25
Oxyrrhynchium hians	<25	50.41 (0.43)	<25	42.22 (1.07)	26.03 (2.38)	46.46 (0.91)
Paraleucobryum longifolium	<25	27.34 (2.34)	<25	<25	<25	<25
Plagiomnium affine	<25	41.79 (1.93)	<25	<25	<25	<25
Plagiomnium cuspidatum	39.13 (1.89)	39.44 (1.50)	26.49 (2.68)	97.60 (0.44)	<25	86.33 (0.69)
Plagiomnium rostratum	26.01 (2.75)	60.72 (2.55)	<25	44.99 (0.98)	28.68 (3.62)	36.26 (1.54)
Plagiomnium undulatum	35.77 (1.26)	33.42 (0.81)	<25	26.07 (0.80)	29.36 (1.13)	43.49 (0.50)

Pleurozium schreberi	61.85 (2.78)	93.41 (1.42)	41.15 (5.11)	37.25 (3.46)	<25	31.65 (2.60)
Pohlia nutans	<25	<25	<25	<25	<25	<25
Polytrichastrum formosum	<25	34.69 (3.54)	<25	<25	<25	28.85 (3.59)
Porella platyphylla	31.89 (4.11)	79.22 (2.61)	48.22 (3.54)	83.33 (0.91)	48.94 (1.93)	64.37 (2.13)
Pseudoleskeella nervosa	68.43 (0.42)	75.64 (0.19)	<25	<25	38.94 (1.48)	43.69 (1.56)
Pseudoscleropodium purum	<25	34.16(0.85)	<25	<25	<25	<25
Rhytidiadelphus squarrosus	<25	<25	<25	<25	<25	<25
Rhytidium rugosum	32.34 (5.29)	56.50 (3.04)	<25	36.29 (2.85)	<25	25.80 (3.09)
Schistidium crassipilum	<25	33.32 (1.92)	<25	<25	<25	<25
Syntrichia ruralis	<25	<25	<25	<25	<25	<25
Thamnobryum alopecurum	29.98 (1.68)	57.12 (0.64)	<25	26.75 (3.98)	<25	<25
Thuidium assimile	<25	<25	<25	29.18 (0.90)	<25	<25

B

Species	HeLa		A2780		T47D	
	10 µg/ml	30 µg/ml	10 µg/ml	30 µg/ml	10 µg/ml	30 µg/ml
Abietinella abietina	30.00 (1.15)	38.62 (1.59)	<25	<25	42.99 (2.04)	48.97 (1.56)
Amblystegium serpens	61.93 (0.99)	70.78 (0.50)	53.46 (1.64)	65.35 (1.89)	70.15 (3.29)	74.76 (2.60)
Anomodon viticulosus	27.04 (2.84)	49.35 (2.17)	32.35 (1.92)	53.87 (0.78)	<25	36.32 (1.28)
Atrichum undulatum	59.93 (0.41)	76.26 (0.53)	37.78 (1.66)	64.28 (1.12)	64.11 (3.12)	65.26 (3.91)
Barbula unguiculata	65.46 (2.74)	75.11 (1.58)	<25	47.47 (3.34)	44.20 (2.82)	53.16 (1.75)
Brachytheciastrum velutinum	34.43 (2.61)	55.09 (3.77)	< 25	61.29 (2.06)	34.43 (5.81)	51.51 (3.12)
Brachythecium rutabulum	51.95 (2.91)	53.89 (0.62)	<25	35.30 (1.34)	46.79 (1.16)	54.92 (0.86)
Bryum argenteum	36.11 (1.79)	54.52 (1.09)	<25	<25	35.95 (3.09)	41.26 (2.91)
Bryum caespiticium	48.64 (3.12)	59.57 (1.50)	<25	<25	28.58 (3.37)	48.17 (4.64)
Bryum moravicum	46.72 (0.53)	62.09 (0.82)	27.34 (1.01)	48.22 (0.85)	40.64 (1.54)	59.69 (0.72)
Calliergonella cuspidata	<25	32.79 (1.46)	<25	<25	39.02 (2.24)	49.49 (1.51)
Ceratodon purpureus	30.67 (1.09)	42.00 (1.63)	<25	35.18 (0.90)	<25	28.48 (2.32)
Cirriphyllum piliferum	<25	28.32 (1.82)	<25	<25	<25	<25
Climacium dendroides	56.79 (0.91)	64.89 (0.64)	<25	<25	55.46 (0.672)	57.16 (1.58)
Dicranum tauricum	33.14 (2.08)	51.60 (1.32)	<25	48.94 (1.07)	35.38 (0.82)	54.93 (0.62)
Encalypta streptocarpa	54.46 (1.51)	72.90 (0.77)	73.72 (1.05)	80.12 (1.13)	33.22 (4.37)	33.27 (5.22)
Funaria hygrometrica	48.44 (0.86)	62.88 (0.69)	25.66 (1.05)	51.06 (1.48)	46.44 (0.57)	53.18 (1.01)
Homalothecium lutescens	<25	<25	<25	<25	28.74 (0.46)	30.64 (0.91)
Homalothecium philippeanum	46.93 (2.67)	73.77 (0.37)	33.60 (0.99)	74.93 (0.44)	63.90 (2.95)	62.51 (0.86)
Hygroamblystegium tenax	36.99 (2.53)	43.86 (2.68)	<25	<25	49.19 (3.56)	55.28 (0.94)
Leskea polycarpa	<25	31.32 (0.81)	<25	35.02 (0.66)	<25	37.07 (1.98)
Leucodon sciuroides	42.48 (1.54)	61.74 (1.30)	<25	<25	28.88 (3.49)	39.63 (2.96)
Neckera besseri	69.13 (0.87)	83.28 (0.50)	<25	76.48 (1.15)	50.07 (2.45)	68.26 (1.66)
Orthotrichum diaphanum	40.19 (0.83)	51.75 (0.72)	25.04 (0.68)	50.65 (1.23)	35.43 (0.83)	40.61 (1.50)
Oxyrrhynchium hians	25.65 (0.90)	39.79 (0.47)	<25	28.61 (0.80)	34.01 (1.18)	46.64 (0.77)
Paraleucobryum longifolium	78.54 (0.43)	83.93 (2.45)	63.23 (2.11)	78.03 (1.98)	46.84 (1.54)	56.87 (2.33)
Plagiomnium affine	42.41 (0.85)	55.53 (1.18)	<25	42.11 (1.43)	42.49 (1.47)	56.05 (1.15)
Plagiomnium cuspidatum	<25	<25	<25	56.15 (0.88)	<25	36.11 (2.43)
Plagiomnium rostratum	46.52 (1.22)	60.22 (1.40)	43.23 (0.79)	67.06 (0.66)	45.56 (3.22)	54.59 (0.79)
Plagiomnium undulatum	<25	<25	<25	33.21 (0.80)	<25	32.10 (1.17)
Pleurozium schreberi	60.49 (2.81)	74.30 (3.28)	<25	36.89 (3.12)	29.26 (3.08)	43.95 (5.88)

Pohlia nutans	29.51 (1.40)	49.60 (2.16)	<25	<25	<25	<25
Polytrichastrum formosum	<25	34.18 (2.39)	<25	<25	<25	<25
Porella platyphylla	35.69 (2.44)	47.36 (4.64)	<25	41.93 (4.18)	29.33 (5.18)	47.86 (4.02)
Pseudoleskeella nervosa	61.71 (0.93)	71.88 (0.81)	<25	36.28 (2.15)	42.77 (1.08)	45.50 (0.85)
Pseudoscleropodium purum	62.06 (0.87)	70.27 (1.15)	<25	28.01 (0.92)	53.88 (1.34)	54.58 (0.89)
Rhytidiadelphus squarrosus	43.99 (1.02)	53.66 (0.31)	<25	<25	40.31 (0.79)	51.65 (1.21)
Rhytidium rugosum	30.20 (5.18)	39.48 (3.83)	<25	<25	<25	27.52 (4.16)
Schistidium crassipilum	27.52 (1.19)	53.09 (1.22)	<25	72.36 (1.26)	<25	38.36 (5.18)
Syntrichia ruralis	27.05 (0.85)	33.02 (0.39)	<25	<25	30.49 (2.365)	39.25 (1.58)
Thamnobryum alopecurum	34.35 (2.31)	53.87 (1.34)	<25	51.91 (1.46)	<25	<25
Thuidium assimile	43.36 (1.23)	57.09 (0.69)	34.62 (4.58)	58.86 (2.92)	65.7 (2.40)	56.12 (1.25)

C

**Species**	**HeLa**		**A2780**		**T47D**	
	10 µg/ml	30 µg/ml	10 µg/ml	30 µg/ml	10 µg/ml	30 µg/ml
Abietinella abietina	35.25 (0.83)	52.48 (1.28)	<25	<25	38.22 (1.17)	49.01 (1.96)
Amblystegium serpens	33.19 (2.39)	44.83 (0.78)	<25	26.71 (1.71)	48.58 (2.21)	58.34 (1.53)
Anomodon viticulosus	<25	34.02 (1.32)	<25	<25	<25	27.47 (3.07)
Atrichum undulatum	29.14 (1.63)	41.65 (0.89)	<25	46.14 (1.91)	<25	37.95 (1.76)
Barbula unguiculata	29.68 (2.16)	35.91 (2.38)	<25	<25	<25	27.68 (4.30)
Brachytheciastrum velutinum	<25	32.09 (2.79)	< 25	< 25	27.26 (6.57)	34.70 (5.61)
Brachythecium rutabulum	<25	34.26 (2.34)	<25	<25	<25	34.81 (3.09)
Bryum argenteum	<25	<25	<25	<25	<25	<25
Bryum caespiticium	<25	35.57 (4,31)	<25	<25	<25	<25
Bryum moravicum	<25	37.94 (2.68)	<25	<25	<25	26.51 (4.96)
Calliergonella cuspidata	<25	<25	<25	<25	<25	<25
Ceratodon purpureus	<25	<25	<25	<25	<25	31.86 (1.37)
Cirriphyllum piliferum	28.18 (4.18)	42.07 (3.32)	<25	<25	<25	26.85 (2.86)
Climacium dendroides	<25	<25	<25	<25	<25	27.68 (2.49)
Dicranum tauricum	<25	28.29 (2.10)	<25	<25	33.52 (3.11)	49.97 (2.64)
Encalypta streptocarpa	28.01 (3.29)	39.61 (3.25)	<25	<25	<25	32.50 (3.81)
Funaria hygrometrica	<25	<25	<25	<25	42.27 (1.43)	48.22 (3.83)
Homalothecium lutescens	<25	<25	<25	<25	<25	<25
Homalothecium philippeanum	<25	33.51 (1.19)	<25	28.04 (1.21)	43.32 (2.19)	51.00 (3.50)
Hygroamblystegium tenax	26.66 (2.09)	38.22 (1.10)	<25	<25	52.69 (1.43)	55.03 (1.22)
Leskea polycarpa	25.62 (1.90)	31.09 (2.17)	<25	<25	<25	34.12 (1.90)
Leucodon sciuroides	<25	29.98 (2.41)	<25	<25	<25	<25
Neckera besseri	37.13 (0.79)	41.25 (2.07)	<25	<25	54.50 (3.31)	55.63 (0.79)
Orthotrichum diaphanum	<25	40.79 (2.06)	<25	<25	<25	28.20 (2.09)
Oxyrrhynchium hians	<25	<25	<25	<25	<25	29.53 (0.99)
Paraleucobryum longifolium	<25	<25	<25	<25	<25	<25
Plagiomnium affine	42.04 (0.87)	50.67 (1.78)	<25	26.86 (2.04)	53.30 (1.02)	57.53 (1.97)
Plagiomnium cuspidatum	35.49 (0.88)	46.35 (0.43)	<25	<25	33.53 (0.69)	45.90 (2.67)
Plagiomnium rostratum	40.44 (2.14)	51.65 (1.79)	<25	42.42 (1.42)	33.23 (3.53)	45.84 (1.16)
Plagiomnium undulatum	<25	<25	<25	<25	<25	<25
Pleurozium schreberi	<25	32.99 (3.41)	<25	<25	<25	<25

Pohlia nutans	<25	32.63 (0.99)	<25	<25	<25	<25
Polytrichastrum formosum	<25	<25	<25	<25	<25	<25
Porella platyphylla	<25	41.69 (1.39)	<25	<25	<25	27.66 (4.52)
Pseudoleskeella nervosa	60.51 (0.47)	65.03 (0.40)	<25	26.27 (3.16)	49.89 (1.66)	54.50 (1.17)
Pseudoscleropodium purum	<25	28.06 (1.95)	<25	<25	<25	31.29 (3.19)
Rhytidiadelphus squarrosus	<25	26.56 (1.10)	<25	<25	<25	33.78 (0.84)
Rhytidium rugosum	<25	33.67 (5.71)	<25	<25	<25	<25
Schistidium crassipilum	<25	<25	<25	<25	<25	<25
Syntrichia ruralis	<25	<25	<25	<25	<25	<25
Thamnobryum alopecurum	<25	31.90 (1.29)	<25	<25	<25	<25
Thuidium assimile	<25	<25	<25	34.23 (1.67)	29.78 (3.06)	43.24 (1.17)

D

**Species**	**HeLa**		**A2780**		**T47D**	
	10 µg/ml	30 µg/ml	10 µg/ml	30 µg/ml	10 µg/ml	30 µg/ml
Abietinella abietina	<25	<25	<25	<25	<25	30.04 (2.98)
Amblystegium serpens	<25	<25	<25	<25	27.91 (3.83)	35.73 (1.85)
Anomodon viticulosus	<25	<25	<25	<25	<25	<25
Atrichum undulatum	33.37 (1.95)	36.14 (0.87)	<25	<25	<25	<25
Barbula unguiculata	27.92 (5.90)	27.40 (2.78)	<25	<25	<25	<25
Brachytheciastrum velutinum	34.90 (3.94)	34.68 (3.51)	< 25	< 25	38.53 (3.42)	38.46 (5.66)
Brachythecium rutabulum	<25	<25	<25	<25	<25	<25
Bryum argenteum	<25	<25	<25	<25	<25	<25
Bryum caespiticium	<25	<25	<25	<25	<25	<25
Bryum moravicum	<25	<25	<25	<25	<25	<25
Calliergonella cuspidata	<25	<25	<25	<25	<25	<25
Ceratodon purpureus	<25	<25	<25	<25	<25	<25
Cirriphyllum piliferum	<25	32.27 (2.64)	<25	<25	<25	<25
Climacium dendroides	<25	27.42 (1.19)	<25	<25	27.38 (1.39)	37.52 (1.51)
Dicranum tauricum	29.75 (1.06)	37.11 (1.85)	<25	<25	45.31 (0.69)	47.21 (1.07)
Encalypta streptocarpa	27.05 (5.44)	<25	<25	<25	<25	<25
Funaria hygrometrica	25.11 (2.09)	38.47 (1.11)	<25	<25	35.40 (3.59)	45.16 (1.29)
Homalothecium lutescens	<25	<25	<25	<25	<25	27.60 (2.80)
Homalothecium philippeanum	<25	<25	<25	<25	33.68 (3.49)	41.04 (3.78)
Hygroamblystegium tenax	<25	31.71 (0.58)	<25	<25	37.34 (1.29)	40.38 (1.70)
Leskea polycarpa	<25	<25	<25	<25	<25	<25
Leucodon sciuroides	<25	<25	<25	<25	<25	<25
Neckera besseri	<25	<25	<25	<25	32.28 (1.25)	43.28 (1.31)
Orthotrichum diaphanum	<25	<25	<25	<25	<25	<25
Oxyrrhynchium hians	<25	<25	<25	<25	<25	<25
Paraleucobryum longifolium	<25	<25	<25	<25	<25	<25
Plagiomnium affine	<25	<25	<25	<25	<25	<25
Plagiomnium cuspidatum	<25	<25	<25	<25	<25	27.79 (2.89)
Plagiomnium rostratum	<25	<25	<25	<25	<25	<25
Plagiomnium undulatum	<25	<25	<25	<25	<25	<25
Pleurozium schreberi	<25	<25	<25	<25	<25	<25

Pohlia nutans	<25	<25	<25	<25	<25	<25
Polytrichastrum formosum	<25	<25	<25	<25	<25	<25
Porella platyphylla	<25	<25	<25	<25	<25	<25
Pseudoleskeella nervosa	<25	<25	<25	<25	<25	25.43 (1.09)
Pseudoscleropodium purum	<25	29.89 (1.63)	<25	<25	32.22 (0.68)	40.05 (0.70)
Rhytidiadelphus squarrosus	<25	<25	<25	<25	<25	<25
Rhytidium rugosum	<25	<25	<25	<25	<25	<25
Schistidium crassipilum	<25	<25	<25	<25	<25	<25
Syntrichia ruralis	30.83 (1.58)	41.17 (1.41)	<25	<25	57.42 (1.21)	59.35 (1.19)
Thamnobryum alopecurum	26.26 (1.55)	40.68 (1.46)	<25	<25	<25	<25
Thuidium assimile	<25	<25	<25	<25	32.28 (2.47)	44.58 (3.04)

Table S2. The used lock masses and the charge state mode. The list contains the known background ion listed by the Waters company (https://www.waters.com/webassets/cms/support/docs/bkgrnd_ion_mstr_list.pdf) and permanently presented negative ions from the blank injection.

m/z	charge
593.15761	Positive
149.02332	Positive
391.28429	Positive
285.91063	Negative
307.86064	Negative
112.98628	Negative

Table S3. Parameters of the data dependent acquisition in both charge states

	Full MS	dd-MS^2
Resolution	70,000	17,500
AGC target	3.00E+06	1.00E+05
Maximum IT	100 ms	50 ms
Scan range	80 to 1000 m/z	200 to 2000 m/z
TopN		5
Isolation window		2.0 m/z
stepped (N)CE		nce: 30, 50, 70
Dynamic exclusion		10.0 s

Table S4. Compounds identified by LC-MS

Species	Extract	Identified compounds
Atrichum undulatum	B	hexadecasphinganine, hydroxyoctadecadienoic acid, 18-oxooleate, vernolic acid
Barbula unguiculata	B	hydroxyoctadecadienoic acid, 18-oxooleate, vernolic acid
Bryum argenteum	A	hydroxyoctadecadienoic acid, lupenone, octadecanamide, 18-oxooleate, vernolic acid,
Encalypta streptocarpa	A	azelaic acid, hexadecasphinganine, hydroxyoctadecadienoic acid, 18-oxooleate, phytuberin, vernolic acid
	B	abietic acid, alpha-amylcinnamaldehyde, artemorin, dehydroabietic acid, dehydocostus lactone, dehydromyodesmone, eremanthin, glutinosone, hydroxyoctadecadienoic acid, isodehydrocostus lactone, ivalin, neoabietic acid, oblongolide, 18-oxooleate, pseudoivalin, santamarin, vernolic acid
Neckera besseri	B	azelaic acid, hexadecasphinganine, hydroxyoctadecadienoic acid, 18-oxooleate, vernolic acid
Paraleucobryum longifolium	B	buddledin A, hexadecasphinganine
Pleurozium schreberi	A	hexadecasphinganine, glutinone, hydroxyoctadecadienoic acid, lupenone, mammeisin, 18-oxooleate, phytuberin, vernolic acid
Porella platyphylla	A	azelaic acid, budledin A, hexadecasphinganine, hydroxyoctadecadienoic acid, 18-oxooleate, vernolic acid
